# Testosterone Mediates Reproductive Toxicity in *Caenorhabditis elegans* by Affecting Sex Determination in Germ Cells through *nhr-69*/*mpk-1*/*fog-1*/*3*

**DOI:** 10.3390/toxics12070502

**Published:** 2024-07-10

**Authors:** Ke Meng, Ying-Chi Shi, Wei-Xi Li, Jia Wang, Bei-Jing Cheng, Tian-Lin Li, Hui Li, Nan Jiang, Ran Liu

**Affiliations:** 1 Key Laboratory of Environmental Engineer Medicine Engineering, Ministry of Education, School of Public Health, Southeast University, Nanjing 210009, China; mk199606@163.com (K.M.); 15380021013@163.com (Y.-C.S.); weixili0212@163.com (W.-X.L.); xueerdawa_wj@163.com (J.W.); chengbeijing@seu.edu.cn (B.-J.C.); ltl201735222@163.com (T.-L.L.); xiaohuixxi@163.com (H.L.); 2 School of Biology and Food Engineering, Fuyang Normal University, Fuyang 236037, China

**Keywords:** testosterone (T), *Caenorhabditis elegans*, sex determination, *nhr-69*, benchmark dose (BMD)

## Abstract

Testosterone (T), an environmental androgen, significantly disrupts endocrine systems in wildlife and ecosystems. Despite growing concern over its high levels in aquatic environments, the reproductive toxicity of testosterone and its mechanisms are not well understood. In this study, we investigated the reproductive toxicity and mechanisms of testosterone using *Caenorhabditis elegans* (*C. elegans*) and assessed its ecological toxicity through the benchmark dose (BMD) method. Our results indicate that T concentrations exceeding 0.01 μg/L significantly reduce the brood size, decrease germ cell counts, and prolong the generation time in *C. elegans* as T concentrations increase. Furthermore, to elucidate the specific mechanisms, we analyzed the expression of *nhr-69*, *mpk-1*, and other genes involved in sex determination. These findings suggest that the *nhr-69*-mediated reproductive toxicity of T primarily affects sperm formation and the offspring number by influencing its downstream targets, *mpk-1* and *fog-1*/*3*, which are critical in the germ cell sex-determining pathway. Additionally, this study determined that the 10% lower boundary of the baseline dose (BMDL10) is 1.160 ng/L, offering a more protective reference dose for the ecological risk assessment of T. The present study suggests that *nhr-69* mediates the reproductive toxicity of T by influencing *mpk-1* and *fog-1*/*3*, critical genes at the end of the germ cell sex-determining pathway, thereby providing a basis for establishing reproductive toxicity thresholds for T.

## 1. Introduction

Endocrine-disrupting chemicals (EDCs) are among the most serious environmental threats to soil, water, and biota worldwide [1]. Compared to other EDCs, androgens—a typical environmental steroid hormone—may cause developmental defects and reproductive disorders in some species [2,3]. Studies have confirmed that steroid hormones disrupt endocrine system function in organisms, leading to toxicity [4]. Testosterone (T), known to be the most abundant, active, and crucial natural androgen in the body, is a key subject of our research [5]. Recently, the environmental levels of T have been reported to be significantly higher than those of estrogens, warranting increased attention [6]. Studies indicate that T exposure primarily originates from human sources, livestock manure, and paper mill wastewater emissions [7,8,9]. Reports have indicated that the mass concentrations of T and androstenedione in surface water samples from a specific region in France range from 0.6 to 6.0 ng/L [10]. A detection report from a pasture in Georgia, USA, revealed T concentrations of 15–125 ng/L in surface runoff water and 10–1840 ng/L in pasture runoff water [11]. Additionally, in the Ahar River flowing through Udaipur, India, the steroid hormone concentrations reach up to 1560 ng/L [12]. The concentration of T in the Xiangjiang River is recorded to be 70.4 ng/L. Consequently, exposure to T varies across different environmental media globally [7,8,13], which negatively impacts the reproductive capabilities of various organisms in ecosystems [14].

Ecotoxicological studies have indicated that exposure to T can cause multiple toxic effects in animals and humans. Prolonged exposure to exogenous T in the environment may result in adverse outcomes, including reduced animal populations and diminished ecological diversity [15,16]. For example, exposure to T at 5 μmol/L significantly decreased fecundity, with more pronounced effects following prolonged exposure [17]. A study involving *Parus major* revealed that offspring from females implanted subcutaneously with 0.6 ± 0.015 mg of T experienced delayed hatching and lower survival rates [10]. In mammals, administering a subcutaneous injection of 3 mg of T at days 16–19 of gestation significantly impaired the reproductive function of rat offspring. Furthermore, prolonged exposure to water containing T could reduce both the spawning rate and reproductive capacity of *Daphnia magna* [14]. While existing studies confirm that high concentrations of T induce reproductive toxicity in aquatic organisms, the impact of environmentally relevant concentrations of T on reproductive toxicity remains unclear. Given the rapid rise in widespread environmental exposure, it is crucial to further investigate the mechanisms and effects of reproductive toxicity at environmentally relevant concentrations.

*Caenorhabditis elegans* (*C. elegans*), a free-living nematode, inhabits the soil and primarily feeds on fungi and bacteria. With a life cycle of about 3 days and a reproductive capacity of 300 progeny per hermaphrodite through self-fertilization, *C. elegans*, having up to 80% genomic homology with humans, serves as an excellent model for EDC research [18,19]. Serving as an intermediate between in vitro and mammalian tests, *C. elegans* offers unique advantages in reproductive toxicity studies due to its efficacy in conducting toxicity experiments [19,20]. For example, assessments of the reproductive capacity of nematodes can be conducted by measuring offspring numbers, germ cell and sperm cell counts, and generation times [21]. Additionally, evaluating the development of the nematode’s gonad can be achieved by analyzing the gonadal developmental stages and malformations [22]. Meanwhile, *nhr-69* is a member of the nuclear receptor family of proteins in *C. elegans*, which is homologous to human nuclear receptor (AR), which is activated by binding androgenic hormones [23,24]. Therefore, *nhr-69* may have an important role in the reproductive process of *C. elegans*. 

This study used *C. elegans* to explore the reproductive toxicity of exogenous testosterone (T) at environmentally relevant concentrations and its underlying mechanisms, aiming to predict the effects of T on the reproduction of environmental organisms. The findings suggest that environmental T exposure can impair reproductive functions and gonad development in *C. elegans*, likely through mechanisms involving the gene *nhr-69*. Concurrently, we analyzed experimental data to establish low-dose reproductive toxicity thresholds using the Benchmark Dose (BMD) method. In conclusion, these results guide research into the mechanisms of T toxicity and offer a theoretical foundation for ecological risk assessments of T using BMD-derived safety data, informing the development of related legislation.

## 2. Materials and Methods

### 2.1. Experimental Nematodes and Cultured Conditions

Wild-type N2 and mutant *nhr-69* (OK1926) strains were obtained from the Caenorhabditis Genetics Center, University of Minnesota, USA. Nematodes were cultured on nematode growth medium (NGM) plates inoculated with *Escherichia coli* (OP50) at a constant 20 °C in a biochemical incubator. To achieve synchronous development, the eggs were extracted from hermaphrodites using nematode lysate and incubated at 20 °C overnight to yield synchronously developed L1 larvae for subsequent experiments.

### 2.2. Preparation and Exposure of the Toxic Solution

Testosterone (from Wanqing, Nanjing, China) was dissolved in DMSO to prepare a 1 g/L stock solution. Based on environmental concentrations, we extrapolated this to set environmentally relevant groups at concentrations of 0.001 μg/L, 0.01 μg/L, 0.1 μg/L, 1 μg/L, and 10 μg/L, using M9 solution as the control. One day prior, 40 μL of OP50 bacterial solution was added to each 35 mm sterilized Petri dish, spread evenly, and then incubated. T at concentrations of 0.001 μg/L, 0.01 μg/L, 0.1 μg/L, 1 μg/L, and 10 μg/L, along with M9 for the control group, were added to 200 μL of sterilized venom and dried on an ultra-clean workbench. Following synchronization, the hatched L1 larvae were centrifuged at 3000 rpm for 1 min. Subsequently, 10 μL of this larvae solution was added to each sterilized Petri dish and incubated for 48 h.

### 2.3. Brood Size

After exposure to treatment T, the L1 nematodes were individually transferred to fresh NGM petri dishes, each containing a drop of 2.5 μL OP50 bacteria solution. The petri dishes were rotated every 24 h, and the nematodes were monitored until they ceased egg-laying. The number of offspring for each nematode was observed and recorded, with data collected from 20 nematodes per concentration group.

### 2.4. Generation Time Determination

A new NGM was added to 2.5 μL OP50 bacterial solution in the center. Following exposure to treatment T, nematodes from each concentration were transferred to fresh NGM Petri dishes. The time at which adult nematodes laid their first egg was recorded as T1. Subsequently, the time between the egg developing into an adult and the laying of its first egg was recorded as T2, and the interval (T2−T1) represented the generation time of the nematodes. In each group, data were collected from 20 nematodes.

### 2.5. Gonadal Developmental Stages

The gonad development stages were classified into five categories based on the methodology described by Craig et al. [25], and divided into 5 developmental stages. Following T exposure, the nematodes in each concentration group were rinsed three times with M9 solution and anesthetized using 0.025 mmol/L levamisole hydrochloride. The gonadal development stages of each nematode were detected and captured under a microscope (Olympus, Tokyo, Japan). Each concentration group included observations of 100 nematodes.

### 2.6. Germ Cell and Sperm Cell Count

After exposure, we washed the nematodes from both the exposure and control groups off the petri dishes using M9 buffer and transferred them to non-toxic NGM petri dishes coated with OP50. We then cultured them for 4 h. We transferred nematodes to a glass slide pre-treated with one drop of M9 buffer and rinsed them three times with 90% ethanol, ensuring no nematodes remained on the slide. Once the treated worms were dry and fixed, we added 20 μL of DAPI dye solution (Sigma-Aldrich, St. Louis, MO, USA) and placed coverslips on the glass slides [26,27]. Subsequently, we stained the nematodes in a controlled environment. It is crucial to perform all the above procedures in dark conditions. Finally, we observed the blue fluorescence using a fluorescence microscope Olympus, Tokyo, Japan). We observed and counted the number of germ and sperm cells, and documented the results with photographs. We recorded data from 20 nematodes in each group.

### 2.7. Molecular Docking

The amino acid sequences of NHR-69 and MPK-1 were obtained based on the National Center for Biotechnology Information (NCBI) https://www.ncbi.nlm.nih.gov/ (accessed on 20 July 2023), and the amino acid sequences of NHR-69 and MPK-1 were used to predict the tertiary structure of the NHR-69 and MPK-1 proteins; in addition, Phyre2 http://www.sbg.bio.ic.ac.uk/phyre2/ (accessed on 21 July 2023) was used to predict the tertiary structure of the NHR-69 and MPK-1 proteins online, and the molecular docking of NHR-69 and MPK-1 was performed by SYBYL-X 2.0 software to explore the binding capacity of the two proteins in the tertiary structure.

### 2.8. Quantitative Real-Time PCR (qRT-PCR) Was Used for Expression Analysis

After exposure, we extracted *C. elegans* RNA by using Trizol (Vazyme, Nanjing, China), and the concentration of the obtained RNA was measured with the Nanodrop 1000 spectrometer (Thermo Scientific, Waltham, USA) at a OD260/280 ratio. After cDNA synthesis, real-time PCR was performed by using the SYBR Green Master Mix (Vazyme, Nanjing, China). The relative expression levels of the target genes were measured by the 2^−ΔΔCt^ relative quantitative method [25]. We summarize the real-time PCR primer sequences for the target genes in Table S1. 

### 2.9. Fitting of Benchmark Dose for Reproductive Toxicity

The reproductive toxicity indicators (brood sizes, generation time, the number of cells, and delayed gonadal development) were included to calculate the BMD and BMDL of T using 10% of the benchmark response (BMR). To obtain the best-fit model, firstly, the US EPA BMDS 3.12 software was used to explore the dose–response relationship of T exposure in *C. elegans*; the continuous variables included Exponential, Hill, Polynomial Degree, Power, and Linear models, and the dichotomous variables included Dichotomous Hill, Gamma, Logistic, Log-Logistic, Probit, Log-Probit, Multistage, and Weibull models. The most appropriate dose toxicity model was performed to determine the BMD and BMDL based on the BMDS recommendation and Benchmark Dose Software (version 3.1.2), which is available from the US EPA (Washington, DC, USA). 

### 2.10. Statistic Analysis

Data are expressed as means standard error (SEM), and graphs of the means were created using Graph Pad Prism 6. The measures in the groups were normally distributed and the variance was homogeneous between the groups (Shapiro–Wilk). To explore the importance of the differences between concentrations, one-way analysis of variance (ANOVA) was performed using SPSS 24.0. Differences at *p* < 0.05 were considered statistically significant. BMD modeling and analysis were conducted using a modification of Benchmark Dose Modeling Software (BMDS) version 3.12.

## 3. Results

### 3.1. Effect of T on Reproductive Capacity

**Figure 1:**
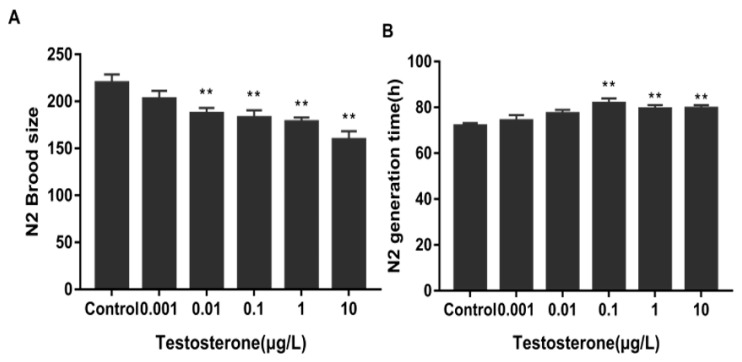
Effects of exogenous testosterone exposure on the fertility of wild-type N2. (**A**) Brood size and (**B**) generation time. Data (mean SEM) are expressed as percentage values compared to the control group, and asterisks indicate significant differences between the exposed and control groups, ** *p* < 0.01.

To investigate the reproductive toxicity of exogenous testosterone in wild-type *C. elegans*, we measured the brood size and generation time. After 48 h of testosterone exposure, the brood size remained stable at very low concentrations (0.001 μg/L), but significantly decreased (*p* < 0.001) with higher concentrations ranging from 0.01 to 10 μg/L. Figure 1A shows that exposure to the highest T concentration (10 μg/L) resulted in a 27.5% reduction in offspring compared to the control group. Significant differences in the generation time were observed among the groups of wild-type N2 nematodes exposed to T concentrations of 0.1 μg/L, 1 μg/L, and 10 μg/L (*p* < 0.05) (Figure 1B).

### 3.2. Effect of T on Gonadal Development

**Figure 2:**
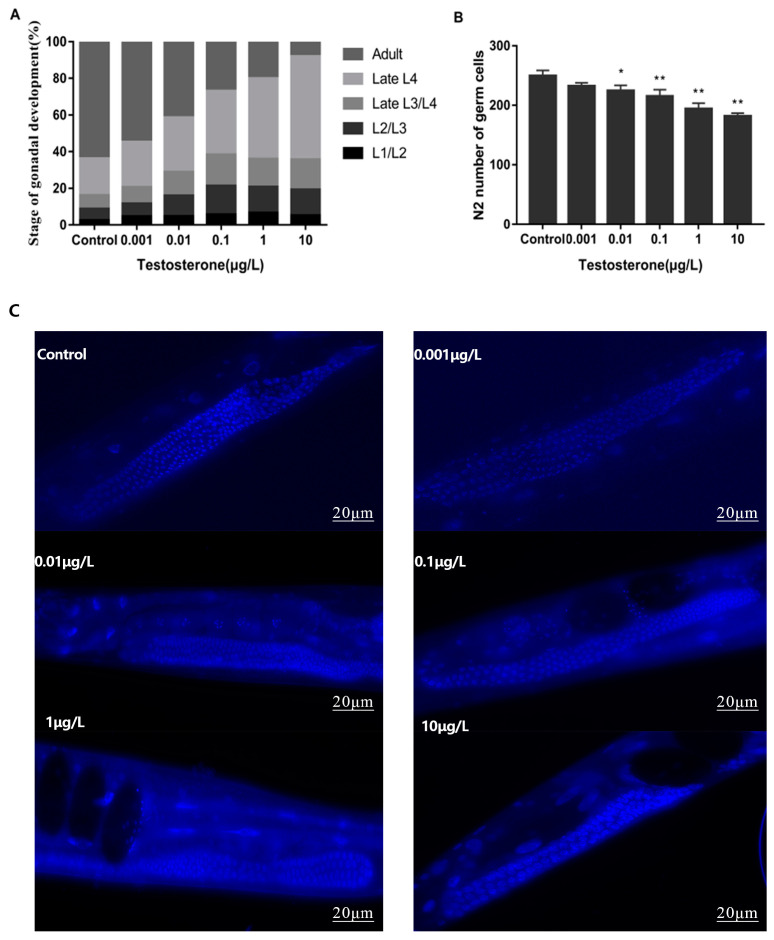
(**A**) shows the percentage of gonadal development in N2 after T exposure (L1, L2, L3 and L4 are the four stages of nematode larvae); (**B**) shows the change in the total germ cell number in N2. Statistically different compared with control; * *p* < 0.05, ** *p* < 0.01, error bars represent standard deviation. (**C**) The images of DAPI staining.

The gonadal development of *C. elegans* was exhibited by the stage of the gonad. In order to investigate the effect of T exposure on the reproductive system of wild-type *C. elegans*, the development of the vulva, gonads, and fertilized eggs of N2 in each dose group was further observed. As shown in Figure 2A, after exposure to T for 48 h and with an increase in the T concentration, compared with the control group, gonad development was gradually inhibited. In a word, the proportion of adult nematodes in the control group and each concentration group decreased from 63.82% ± 16.37% (control group) to 8.00% ± 2.03% (10 μg/L). 

We next focused on its effect on the number of germ cells. After N2 was exposed to exogenous environmentally relevant concentrations of T for 48 h from the L1 stage, DAPI was used to stain each dose group. As shown in Figure 2B, compared with the control group, the number of total germ cells reduced significantly at a higher dose than that used in the 0.01 μg/L group (*p* < 0.05). And the number of total germ cells decreased by 27.1% in the 10 μg/L (181.80 ± 19.45) dose group compared with the control group (249.40 ± 23.09, *p* < 0.001). Additionally, with the increase in the exposure concentration, the number of total germ cells decreased gradually, and there was an obvious dose–response relationship (R = 0.518, *p* < 0.01).

### 3.3. MPK-1 Has a Strong Binding Capacity to NHR-69

**Figure 3:**
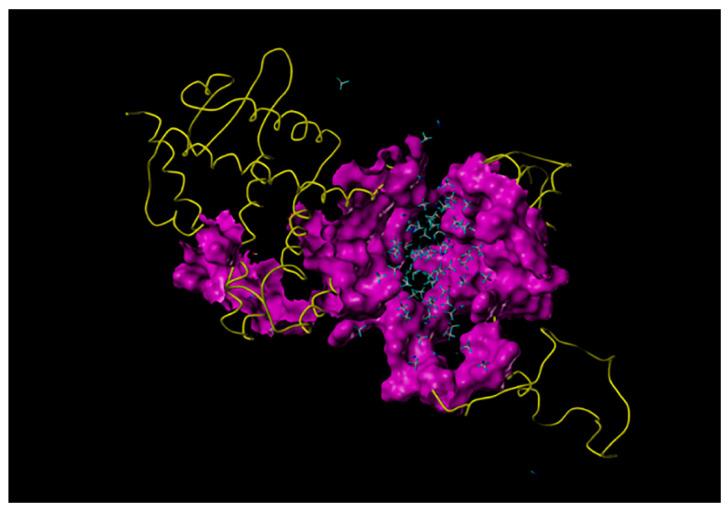
Molecular docking between NHR-69 and MPK-1 proteins. (Yellow indicates a folded structure of an amino acid; purple indicates the binding domain of NHR-69 to MPK-1).

Molecular docking showed that NHR-69 binds strongly to MPK-1, the gene encoding ERK/MAPK. T acts in *C. elegans* to bind to NHR-69. This results in a conformational change of NHR-69 and its dissociation from HSP90 (human heat shock protein HSP90AA1 or HSP90AB1); the separated NHR-69 forms a T-NHR-69 dimer with testosterone, and part of the dimer binds to MPK-1 in the cytoplasm (Figure 3).

### 3.4. Effect of T on Expression of nhr-69 Receptor and Related Sex-Determining Genes 

**Figure 4:**
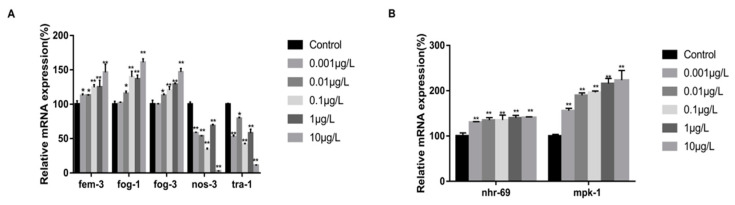
Effect of T exposure on the expression of wild-type *C. elegans*-related genes. (**A**) Key genes of sperm–egg transformation pathway. (**B**) *nhr-69* and *mpk-1*. Data (mean SEM) are expressed as percentage values compared to the control group and asterisks indicate significant differences between the exposed and control groups, * *p* < 0.05, ** *p* < 0.01.3.5. The Reproductive Toxicity of T Was Further Explored on the nhr-69 (OK1926) Mutant Strain.

After 48 h of T exposure, real-time PCR analysis was conducted on *nhr-69*, *mpk-1*, and key genes that contribute to spermatogenesis during sex determination in germ cells, including *nos-3*, *fem-3*, *tra-1*, *fog-1*, and *fog-3*. As shown in Figure 4A, compared with the control group, the expression levels of genes conducive to spermatogenesis (*fem-3*, *fog-1*, *fog-3*) increased significantly with the increase in the exposure concentration, while the expression levels of *nos-3* and *tra-1* decreased. Noticeably, when the exposure concentration reached 0.01 μg/L, the expression levels of all genes except nos-3 and tra-1 were significantly higher than those of the control group. Meanwhile, as shown in Figure 4B, compared with the control group, the gene expression of N2 *C. elegans* in each concentration group increased significantly (*p* < 0.05). As the concentration of T increased, the expression levels of *nhr-69* and *mpk-1* became higher and higher, ranging from 1.46 (*nhr-69*) to 2.23 (*mpk-1*) as the exposure concentration reached 10 μg/L. To sum up, our results indicate that the reproductive toxicity induced by exogenous T exposure presents a high correlation with *nhr-69*, and that exposure to T affected the sex determination process of wild-type *C. elegans*. These results demonstrate that the expression of each gene altered significantly at the exposure concentration of 0.001–10 μg/L, and that the most significant changes were observed at the exposure dose of 10 μg/L.

**Figure 5:**
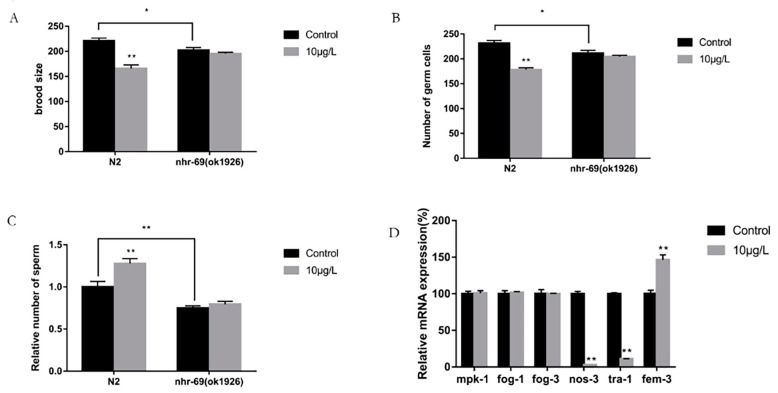
Effects of exogenous testosterone exposure on N2 and *nhr-69* (ok1926). (**A**) Brood size, (**B**) number of germ cells, (**C**) relative number of sperm, and (**D**) the expression of genes related to *nhr-69* (ok1926). Data (mean SEM) are expressed as percentage values compared to the control group, and asterisks indicate significant differences between the exposed and control groups, * *p* < 0.05, ** *p* < 0.01.

To further confirm the relationship between *nhr-69* and the sex determination of wild-type *C. elegans*, we used an exposure dose of 10 μg/L, which induces more significant toxicity, and analyzed the number of offspring, germ cells, sperm cells, and the gene expression of key genes in the sex determination of germ cells in the *nhr-69* (ok1926) mutation. As shown in Figure 5A, the number of offspring in the N2 exposure group decreased by about 25% compared with the control group (*p* < 0.01), while in the *nhr-69* (ok1926), the mutation exposure group did not significantly change compared with the control group (*p* > 0.05). 

After exposure to T, the total number of germ cells in N2 and the *nhr-69* (ok1926) mutation were counted by DAPI staining, and the results of the germ cell count were similar to those of the offspring. As shown in Figure 5B, compared with the control group, the number of germ cells in the N2 exposure group was significantly decreased (*p* < 0.01). By contrast, between the exposed and control groups, the difference in the number of germ cells in the *nhr-69* (ok1926) mutation was not significant. 

By performing the same operation as above, we calculated the number of sperms in each group. The results of the N2 sperm count showed that the number of sperms in the exposed group was significantly higher than that in the control group (*p* < 0.01), reaching about 1.3 times that of the control group. Meanwhile, for the *nhr-69* (ok1926) mutation, there was no significant change in the exposed group compared with the control group (Figure 5C). Simultaneously, when comparing N2 to *nhr-69* (ok1926) and the brood size, number of germ cells and sperm cells in the control group, we found that the results were significantly decreased.

Real-time PCR analysis was used to detect the gene expression of the *nhr-69* mutation. As shown in Figure 5D, the results indicated that the expression levels of *mpk-1* and *fog-1*/*3* in the *nhr-69* (ok1926) mutation did not significantly change compared with the control group at the exposure dose of 10 μg/L (*p* > 0.05), while the expression levels of the *nos-3*, *tra-1* and *fem-3* genes were still significantly changed compared with the control group (*p* < 0.05).

From the above result, we found that the effect of exogenous T exposure on the sex determination of germ cells disappeared in *nhr-69* mutation strains. Therefore, the *nhr-69* gene was involved in the impact that T exposure has on the sex determination of germ cells.

### 3.5. Benchmark Dose Analysis of Toxicity Data of T

According to the reproductive toxicity test results for *C. elegans*, indicators with differences that were statistically significant, had toxicological significance, and had a dose–response relationship were screened for inclusion in the evaluation. The results demonstrated that the brood size, generation time, and the number of germ cells in N2 were determined as endpoints to be included in the benchmark dose (BMD) modeling. Due to all the data being continuous, we selected 10% as a benchmark response (BMR) to estimate the BMD and BMDL. The results are shown in Table 1. In the end, by adopting conservative principles, the indicator with the lowest BMDL value was selected as the final result. Therefore, brood size was fitted as the BMDL10 = 1.160 ng/L for the reproductive toxicity of T in *C. elegans*.

## 4. Discussion

Testosterone (T), known for its stability and high lipophilicity, is widespread in both aquatic and soil environments [16,28]. The detection of T in the environment has increasingly been emphasized as a problem in recent years. However, the literature on the adverse environmental effects of this androgen is scarce compared to that on estrogen [29]. Previous studies have shown that exposure to testosterone induces reproductive toxicity, a form of long-term toxicity, in mammals and aquatic animals. However, research into the mechanisms of reproductive toxicity from T exposure appears to be lacking. 

This study used hatched L1-larval nematodes to examine the reproductive toxicity of environmental concentrations of T, as they are known to be highly sensitive to such effects [28,30]. Our findings indicated significant reductions in the brood size and germ cell counts in *C. elegans* after exposure to T (0.01–10 μg/L). The brood size and germ cell counts served as endpoints for assessing the reproductive toxicity in *C. elegans* [31,32,33]. Substances with endocrine-disrupting properties can reduce germ cell numbers and delay gonad development in *C. elegans* [34]. Although T exposure in mammals does not reduce offspring numbers, prenatal exposure in rats can still cause morphological abnormalities in the reproductive systems of their offspring [33]. Additionally, the generation times significantly increased with T concentrations ranging from 0.1 to 10 μg/L. Similar findings were observed in a study on great tits (*Parus major*), where females implanted with approximately 0.6 mg of T experienced delayed offspring hatching and reduced nestling survival [10]. Our study shows that exposure to environmentally relevant T levels can decrease the reproductive capacity and impair gonad development in nematodes, leading to reproductive toxicity. We focused on the reproductive toxicity of the mechanisms underlying testosterone, identifying *nhr-69* as a potential factor in reducing the reproductive capacity. 

T needs to bind to its receptor to exert its action. *Nhr-69*, homologous to the human androgen receptor (AR), is a nuclear hormone receptor and transcription factor in nematodes that can activate steroidal hormone-binding activity [35,36,37]. The elevated expression of *nhr-69* underscores its pivotal role in T-induced toxicity. However, research on the downstream targets and sex-specific pathways of *nhr-69* following T exposure in *C. elegans* remains limited. *Mpk-1*, a downstream target of *nhr-69*, is integral to hermaphrodite germline development and the male germ cell fate, encoding a mitogen-activated protein (MAP) kinase and an ERK ortholog [23,38]. We assessed the expression of *mpk-1*, a potential target of *nhr-69*, alongside the key genes involved in germline sex determination. Post-T exposure, the *mpk-1*, *fem-3*, *fog-1*, and *fog-3* expression levels were elevated, whereas the *nos-3* and *tra-1* expression levels were reduced. These gene expression changes correlated with phenotypic observations. Notably, in *nhr-69* mutant strains, unlike wild-type *C. elegans*, the difference in the germ and sperm cell numbers between exposed and control groups was insignificant. Additionally, the count of germ and sperm cells in mutant strains consistently fell below that in N2. These findings suggest that testosterone mediates reproductive toxicity via *nhr-69*, influencing sperm and oocyte production and leading to a disproportionate sperm-to-oocyte ratio. In a recent study, it was also observed that the exposure of *C. elegans* to fenitrothion induces toxicity through nhr-69-mediated effects on the meiosis of germ cells [39,40]. We further analyzed the *mpk-1*, *nos-3*, *tra-1*, *fem-3*, and *fog-1*/*3* gene expression in *nhr-69* mutant strains. Compared to N2, only *nos-3*, *tra-1*, and *fem-3* showed activation, with expression levels similar to those in N2. Integrating phenotypic characteristics with gene expression data, our results demonstrate that *nhr-69* mediates reproductive toxicity via the sex determination pathway.

There are approximately 30 genes associated with the sexual fate of germline in *C. elegans* [41,42], and some them were important for our research. In the sex determination pathway, *fog-1* and *fog-3* regulate sperm production by germ cells [43]. However, mutations in either gene result in all germ cells differentiating into oocytes [44]. Both *fog-1* and *fog-3* function, as terminal regulators at the end of the germline sex determination pathway, directly initiate spermatogenesis [45]. *Mpk-1* is likely to function upstream of fog-1 and fog-3, directly enhancing their expression. In the oocyte fate pathway, *nos-3*, a homolog of Drosophila *NANOS*, interacts physically with *FBF-1*. It is essential for hermaphrodite germline development, promoting the transition from sperm to oocyte production by potentially forming a complex with *FBF-1* that regulates *fem-3* mRNA [46]. During development, tra-1 autonomously acts as the terminal regulator of the sex determination pathway, positively influencing all aspects of hermaphrodite somatic sexual differentiation [47]. *Fem-3*, a crucial gene, promotes spermatogenesis by acting upstream of *fog-1* and *fog-3* during sex determination. Mutations in *fem-3* can result in the feminization of the germline [38]. In the regulatory pathway determining the oocyte fate, *nos-3* is positioned upstream of *fem-3*, while *tra-1* is downstream. Additionally, *tra-1* directly regulates both *fog-1* and *fog-3*, inhibiting male development and spermatogenesis and reducing the expression of *fog-1* and *fog-3* [41,47]. Exposing *C. elegans* to testosterone reveals a reproductive toxicity mechanism related to sex determination, potentially causing an abnormal sperm-to-oocyte ratio during this process. Other aquatic organisms have shown similar phenomena; there are reports of *Mosquito fish* being masculine in rivers in the USA and Europe due to potential environmental androgens [48]. In conclusion, *nhr-69*, the receptor for testosterone, plays a crucial role in sex determination, and exposure to testosterone activates *nhr-69*, which subsequently influences spermatogenesis by modulating the *mpk-1*/*fog-1*/*3* pathway; this leads to more sperm than oocytes being produced.

Exposure to organisms can result in varying degrees of harm depending on the outcomes. Since contaminants typically affect multiple indicators, assessing the toxicity of exogenous chemicals requires multiple effect indicators [49]. Our study incorporated various effect indicators into the BMD model to reflect the reproductive toxicity of androgenic testosterone, and analyzed the sensitivity of each indicator by establishing baseline dose–response curves. Furthermore, according to the conservative principle [50], the biological exposure limits for exogenous chemicals, determined using the Benchmark Dose (BMD) method, are considered safer than those derived from traditional non-carcinogenic risk assessment methods. Proposing accurate Benchmark Dose Lower (BMDL) values is crucial for enhancing the reliability of risk assessments. Our results showed that the lowest BMDL10 value, 1.160 ng/L, was associated with the number of offspring, which is a traditional indicator of reproductive toxicity. Recent studies have indicated that the BMDL of T approaches the lower levels of environmental exposure in some regions. For example, one study found that the concentrations of T detected in the surface waters of South Korea were under 0.5 ng/L [51]. However, several separate studies have found a significantly higher concentration than the BDML (1.160 ng/L), with 10 ng/L to 1840 ng/L, in pasture runoff water and wastewater treatment plants [11,52]. This suggests a need for stringent control of waterborne testosterone concentrations near pastures and wastewater treatment plants, as well as the establishment of stricter standards for controlling T exposure in various environments. This is crucial to protect the species diversity within the ecological environment and to generate data for estimating safe human exposure levels to T. 

This research still has several limitations. The toxicological data on environmental T exposure, obtained using *C. elegans*, require further population research to investigate the health risks associated with exposure and its effects. Additionally, the BMD method, being a single model, struggles to characterize complex organism responses. Its selection may overlook model uncertainties and omit valuable information from other models, thus diminishing the reliability of BMD and BMDL estimates. Further research is needed to propose modifications and new methods for the statistical modeling of BMD.

In brief, we detected a difference from the previous sex determination pathway established in *C. elegans*, which was activated on the exposure of *C. elegans* to T. Our results indicated that the *nhr-69*-mediated reproductive toxicity of T affects sperm formation, mainly by affecting *mpk-1*, its downstream target, and *fog-1*/*3*, the most terminal part of the sex determination pathway in germ cells. Additionally, this study marks the first use of BMD to evaluate the reproductive toxicity of T in nematodes, contributing to the ecological risk assessment and management of environmental T.

## 5. Conclusions

Recent studies have shown that exposure to T at environmentally relevant concentrations induces reproductive toxicity in wild-type *C. elegans*, characterized by a reduced brood size, fewer germ cells, delayed gonadal development, and a prolonged generation time. Our further study analyzed the sex determination pathway of germline germ cells, and the results showed that T induced reproductive toxicity through the *nhr-69*/*mpk-1*/*fog-1*/*3* pathway. Moreover, the BMDL10 was 1.160 ng/L for T, providing toxicological data for establishing the relevant limits of reproductive toxicity and performing a comprehensive ecotoxicological evaluation.

## Figures and Tables

**Table 1:** Benchmark dose fitting results of the testosterone-induced reproductive toxicity sensitivity indicators for *C. elegans*.

Indicators	Model	BMD (μg/L)	BMDL (μg/L)	Test 4 (P)	AIC	BMDS Recommendation Notes
Brood size	Hill	4.089	1.160	0.132	561.358	Viable—Lowest AIC BMD/BMDL ratio > 3
Generation time	Hill	10.402	5.079	0.378	301.184	Viable—Lowest AIC
The number of germ cells	Hill	100.696	17.169	0.245	447.468	Viable—Lowest BMDL BMD/BMDL ratio > 3

Abbreviations: BMD: Benchmark response; BMDL: lower value of 95% confidence interval of BMD; Test 4 (P): the result of the goodness-of-fit test; AIC: Akaike’s information criterion.

## Data Availability

The datasets used and/or analyzed during the current study are available from the corresponding author upon reasonable request.

## Supplementary Materials

The following supporting information can be downloaded at: https://www.mdpi.com/article/10.3390/toxics12070502/s1, Table S1: Primers used for real-time RT-PCR.
