# Supplementary material for: Testosterone Mediates Reproductive Toxicity in Caenorhabditis elegans by Affecting Sex Determination in Germ Cells through nhr-69/mpk-1/fog-1/3
Source: Toxics. 2024 Jul 10;12(7):502. doi: 10.3390/toxics12070502 (PMC11281075; doi:10.3390/toxics12070502)
Supplement: Supplementary file 1 [file toxics-12-00502-s001.zip › toxics-3079162-supplementary.pdf]

Table S1. Primers used for real-time RT-PCR.

Gene	Forward（5'-3'）	Reverse（3'-5'）
nhr-69	GCAGAGAAGGTGTACTGACAAGGC	CGAACAAACGAGCAAGTTGGACATC
fem-3	CTTGGAATGCCACCACTGGAAAATG	CCAGTTATGTCGCACCAAATCGTTG
fog-1	CGGTCCGAATGAATCCAGAAGCC	GTTGGAGCACGATGGACAGTAGTAC
fog-3	AACCTTCAGTGGCGTTGAGTTCTC	AGACGAAGACGAAACTTGCTGAGAG
mpk-1	CCAGGAGATGAGCCAGTTTGTGAG	CGCTTCTGCCTCCATTCGTCTG
nos-3	GCAGCCTCAGCAACAACATCAAC	CCTCCTTGTGGCATTCCGTATGG
tra-1	TCGGAGGATTCGGAGATGAAGAGG	TCAGTCTCTACCACCACCACCATC
Y45F10D.4	CGAGAACCCGCGAAATGTCGGA	CGGTTGCCAGGGAAGATGATGAGGC
